# An integrated structural proteomics approach along the druggable genome of *Corynebacterium pseudotuberculosis *species for putative druggable targets

**DOI:** 10.1186/1471-2164-16-S5-S9

**Published:** 2015-05-26

**Authors:** Leandro G Radusky, Syed Shah Hassan, Esteban Lanzarotti, Sandeep Tiwari, Syed Babar Jamal, Javed Ali, Amjad Ali, Rafaela Salgado Ferreira, Debmalya Barh, Artur Silva, Adrián G Turjanski, Vasco AC Azevedo

**Affiliations:** 1 Departamento de Química Biológica, Facultad de Ciencias Exactas y Naturales, Universidad de Buenos Aires, Pabellón II, Buenos Aires C1428EHA, Argentina; 2 INQUIMAE/UBA-CONICET, Facultad de Ciencias Exactas y Naturales, Universidad de Buenos Aires, Pabellón II, Buenos Aires C1428EHA, Argentina; 3 PG Program in Bioinformatics, Institute of Biological Sciences, Federal University of Minas Gerais, Belo Horizonte, Minas Gerais, Brazil; 4 Department of Chemistry, Kohat University of Science and Technology (KUST), KPK, Pakistan; 5 Department of Industrial Biotechnology, Atta-ur-Rahman School of Applied Biosciences, National University of Sciences & Technology (NUST), Islamabad, Pakistan; 6 Department of Biochemistry and Immunology, Institute of Biological Sciences, Federal University of Minas Gerais, Belo Horizonte, Minas Gerais, Brazil; 7 Centre for Genomics and Applied Gene Technology, Institute of Integrative Omics and Applied Biotechnology (IIOAB), Nonakuri, Purba Medinipur, West Bengal, India; 8 Institute of Biological Sciences, Federal University of Pará, Belém, Para, Brazil

**Keywords:** *Corynebacterium pseudotuberculosis *(Cp), Druggable genome, Structural proteomics approach, Putative globally/conserved druggable/bindable targets, Caseous lymphadenitis

## Abstract

**Background:**

The bacterium *Corynebacterium pseudotuberculosis *(Cp) causes caseous lymphadenitis (CLA), mastitis, ulcerative lymphangitis, and oedema in a number of hosts, comprising ruminants, thereby intimidating economic and dairy industries worldwide. So far there is no effective drug or vaccine available against Cp. Previously, a pan-genomic analysis was performed for both biovar *equi *and biovar *ovis and a *Pathogenicity Islands (PAIS) analysis within the strains highlighted a large set of proteins that could be relevant therapeutic targets for controlling the onset of CLA. In the present work, a structural druggability analysis pipeline was accomplished along 15 previously sequenced Cp strains from both biovar *equi *and biovar *ovis*.

**Methods and results:**

We computed the whole modelome of a reference strain Cp1002 (NCBI Accession: NC_017300.1) and then the homology models of proteins, of 14 different Cp strains, with high identity (≥ 85%) to the reference strain were also done. Druggability score of all proteins pockets was calculated and only those targets that have a highly druggable (HD) pocket in all strains were kept, a set of 58 proteins. Finally, this information was merged with the previous PAIS analysis giving two possible highly relevant targets to conduct drug discovery projects. Also, off-targeting information against host organisms, including *Homo sapiens *and a further analysis for protein essentiality provided a final set of 31 druggable, essential and non-host homologous targets, tabulated in **table S4**, additional file 1. Out of 31 globally druggable targets, 9 targets have already been reported in other pathogenic microorganisms, 3 of them (3-isopropylmalate dehydratase small subunit, 50S ribosomal protein L30, Chromosomal replication initiator protein DnaA) in *C. pseudotuberculosis*.

**Conclusion:**

Overall we provide valuable information of possible targets against *C. pseudotuberculosis *where some of these targets have already been reported in other microorganisms for drug discovery projects, also discarding targets that might be physiologically relevant but are not amenable for drug binding. We propose that the constructed *in silico *dataset might serve as a guidance for the scientific community to have a better understanding while selecting putative therapeutic protein candidates as druggable ones as effective measures against *C. pseudotuberculosis*.

## Background

Efforts to find new bacterial drug and/or vaccine targets are becoming indispensable due to the antimicrobial resistance, rapid loss of effectiveness in antibiotic treatment and the quantitative emergence of multi-resistant microbial strains that pose a global challenge and threat. *Corynebacterium pseudotuberculosis *(Cp) is a pathogen of great veterinary and economic importance, since it affects a broad spectrum of animal livestock worldwide, mainly sheep and goats, as well as mammals in numerous Asiatic, Arabic and African countries, North and Latin America and Australia [1]. *C. pseudotuberculosis *is a Gram-positive, facultative intracellular and pleomorphic organism; it possesses fimbriae but is non-motile in nature [2]. The *rpoB *gene analysis for CMNR group of bacteria (*Corynebacterium*, *Mycobacterium*, *Nocardia *and *Rhodococcus*), which has a great medical, veterinary and biotechnological importance, has shown a close phylogenetic relationship [3]. A number of pathogenic strains from a wide range of hosts have already been sequenced, demonstrating the importance of this microorganism [3]. The pathogen infects goat and sheep populations (biovar *ovis*), causing caseous lymphadenitis (CLA), a chronic contagious disease with abscess formation in superficial lymph nodes and subcutaneous tissues. Biovar *equi*, on the other hand, infects lung, kidney, liver and spleen in higher mammals like cow, camel, buffalo etc. thereby, threatening the life of herd animals [2,4]. There are few reports in humans of symptoms similar to lymphadenitis abscesses, caused by an occupational exposure to the infected animals [5-7]. Bearing in mind the medical importance of *C. pseudotuberculosis *due to a lack of efficient medicines, here, we have made an effort and applied a computational strategy to search for new therapeutic molecular targets from this bacterium.

Homology modelling is a widely used technique that has proved good results to expand structural space of pathogens [8,9]. We have designed and implemented a protein structure prediction pipeline using homology modelling based on Martí-Renom methodologies [10]. The pipeline was applied to the randomly selected Cp1002 genome as a reference strain; the genomes of other strains were modelled using a mutation procedure, the sequences that present homology in both, i.e., the core genome, were already modelled in the reference strain Cp1002.

The main purpose of this study is to offer information based on recently reported structure-based prediction of protein druggability that might be valuable for target selection in drug design projects. Druggability is a concept used to describe the ability of a given protein to bind a drug-like molecule, which in turn modulates its function in a desired way. Purely, from a structural point of view, it is the likelihood that small drug-like molecules bind a given target protein with high affinity (< 1µM), a concept also referred to "bind ability". Early attempts to determine the druggable genome of an organism were based on counting the number of targets belonging to known druggable domains that have yielded values in range of 10-14% for the human genome. According to our knowledge, a structural druggability assessment for the *C. pseudotuberculosis *multi-strain proteome was never performed before.

Recently, we developed a fast method for druggability prediction based on the open source pocket detection code "fpocket", which combines several physicochemical descriptors to estimate the pocket druggability and that can be used on a genomic scale [11]. Druggable proteins should have a pocket with suitable features that enable binding of a drug-like compound. After computing proteins that remains druggable along the 15 Cp genomes, 58 target candidates were selected.

The Cp genome has been reported to include seven putative pathogenicity islands (PAIS) [12], which contain several classical virulence factors, including genes for fimbrial subunits, adhesion factors, iron uptake and secreted toxins. Additionally, all of the virulence factors in the islands have characteristics that indicate the phenomenon of horizontal gene transfer. The importance of our dataset is enhanced with the emerged information from the literature regarding the PAIS, pan-genome and also with the pan-modelome strategies for target selection [38].

## Methods

### General concept

**Figure 1:**
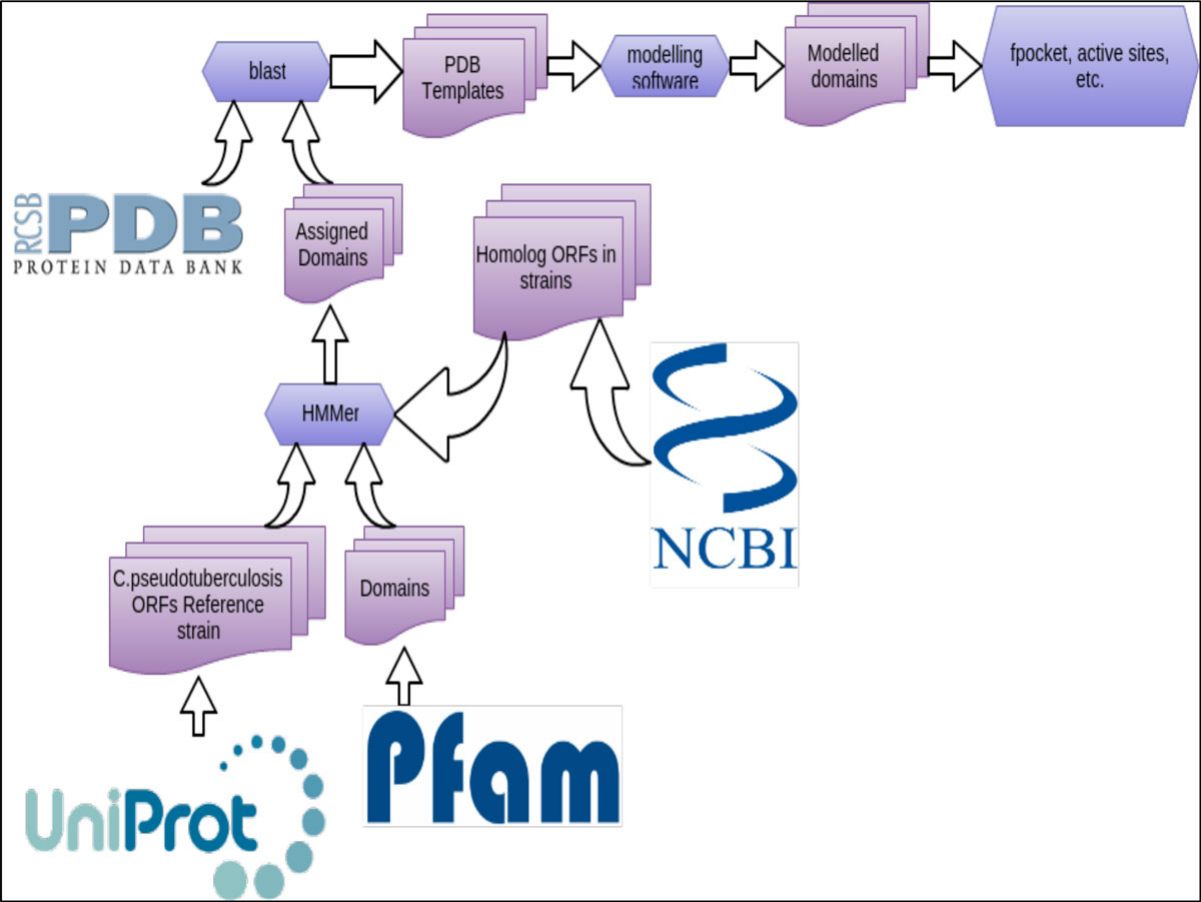
**A general sketch of the pipeline**. All outputs, steps and summaries are available for download purpose and later analyses; links are available in supplementary material.

The druggability analysis pipeline consisted of the following steps (Figure 1). The Open Reading Frame (ORFs) sequences of *C. pseudotuberculosis *were obtained from the GenBank database [13,14]. All ORFs were then analysed with the HMMer software and the structural domains were assigned. Then, each ORF was used to perform a BLAST search against the Protein Data Bank to determine which structure(s) will be used as template(s) to perform homology modelling of the ORFs or computed domains. For all the 3D modelled structures, a set of structural properties were computed, including: i) the Druggability Score (DS) for each pocket, ii) the active site residues (if available) according to the template structures, iii) the conserved or family relevant residues. This information was later combined with the essentiality criteria and the previously related pathogenicity information present in the literature. A detailed description of the programs and databases used to perform each of the above mentioned pipeline step is given below in detail (Figure 1).

### Initial dataset construction

All ORFs or possible proteins for all the strains of Cp were obtained by downloading the information available at the NCBI database (ftp://ftp.ncbi.nih.gov/genomes/Bacteria). The randomly selected strain used as a reference genome for further calculations is Cp1002, according to recent work by Hassan *et al*., 2014 [38]. The Cp1002 genome has 2097 reported ORFs.

### Pfam domain assignment

All the ORFs in the reference proteome were analysed with the HMMer program and were later assigned the Pfam families or domains, leading to a total of 2455 domains assignments from Pfam-A entries and 509 ORFs with no domain assigned. However, as expected, more than one ORF can be assigned to the same domain. Thus, considering this information, a total of 1327 unique (i.e. different) domains were assigned to a whole Cp reference genome. On average, the Cp reference genome has 1.87 domains per ORF and 1.58 unique domains per ORF.

### Generation of structural homology-based models

The strain Cp1002 was used a basis for the structural study. For each sequence in this strain, several models were built using the following procedures: First, a PSI-Blast [15] search was performed against UniRef50, using 3 iterations and an *e.value *threshold of 0.0001, in order to compute a checkpoint that will be used as a profile for the target sequence. Second, PSI-Blast search is restarted using the aforementioned checkpoint against a template library. The template library consisted of all sequences from every individual protein chain in the Protein Data Bank (PDB), grouped at 95% sequence identity threshold using CD-HIT [16]. Afterward, the target-template alignments were computed in the last step, with an *e-value *of ≤ 0.00001 to build the model structures using the MODELLER software [17,18]. For each target-template alignment, ten different target models were built, and their quality measures were assessed using the GA341 [19,20] and QMEAN methods [21]. Only the models with GA341 score ≥ 0.7 (score for the reliability of a model derived from statistical potentials) [22], were retained. A reliable model has a probability of correct fold larger than 95% and coverage of over 50%. Every sequence belonging to the other 14 strains were compared to strain Cp1002 using BLASTp. For each sequence that gives a hit with sequence identity above 85%, a mutation methodology was applied on each amino acid substitution using MODELLER between this sequence and the sequence belonging to Cp1002.

### Structural assessment of druggability

**Figure 2:**
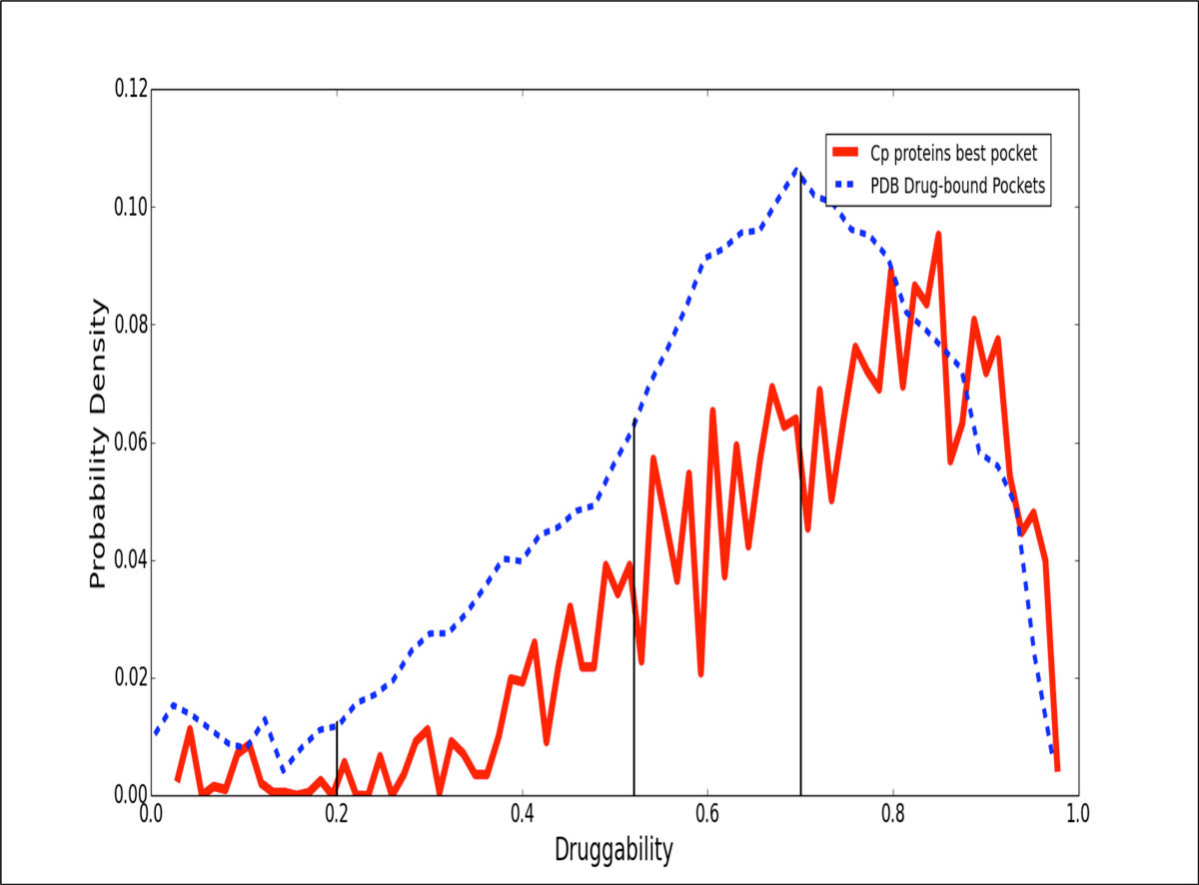
**Histogram of druggability score (DS)**. All ligand-bound structures in the PDB (blue pointed line) and all the modeled structures of the *C. pseudotuberculosis *genome (red line) are represented in the histogram. The scores were computed using the fpocket program for all pockets present in all unique proteins in the PDB, which were crystallized in complex with a drug-like compound. A Gaussian fit of the data made to define these sets was performed in Radusky et al., 2014 [11]. The sets are: non-druggable proteins (ND; DS ≤ 0.2), poorly druggable (PD; 0.2 < DS ≤ 0.5), druggable (D; 0.5 < DS ≤ 0.7), and highly druggable (HD; DS ≥ 0.7). The red line is the distribution of DS over all the models built in this work.

Structural druggability of each modelled and potential target was assessed by determining and characterizing the ability of putative pockets to bind a drug-like molecule by using the fpocket program and the recently developed Drug Score (DS) [23]. Briefly, the method is based upon Voronoi Tessellation algorithm to identify pockets and computes suitable physicochemical descriptors (polar and apolar surface area, hydrophobic density, hydrophobic and polarity score) that are combined to yield the DS, which ranges between 0 (non-druggable, ND) to 1 (highly druggable, HD). In this work we separated the druggability score in four sets: non-druggable proteins (ND; DS ≤ 0.2), poorly druggable (PD; 0.2 < DS ≤ 0.5), druggable (D; 0.5 < DS ≤ 0.7), and highly druggable (HD; DS ≥ 0.7). This distribution based on our previous study where we computed the druggability score for all pockets present in all unique proteins in the PDB, which were crystallized in complex with a drug-like compound [11]. In Figure 2, we compare the druggability distribution of all structures of Cp genome built in this work. Although the distribution has a small shift to higher values; we use the same bounds to define the sets of druggable proteins (Figure 2).

Extending this information along the different strains, those structures labelled as HD in the reference strain were analysed in the other ones. A protein target, which remained druggable in all the strains, was classified as Globally Druggable (GD), also, a protein druggable in all the virulent strains is classified as Virulent Druggable (VD). The GD, obviously, remained a subset of VD.

### Active site identification

In order to identify the active site pocket, our pipeline implements two different analyses that rely on; i) the information from the CSA, and ii) a Pfam position site, as the importance criteria.

The data from CSA was downloaded from http://www.ebi.ac.uk/thornton-srv/databases/CSA/ that consisted of a list of PDB_IDs linked to a number of residues, which comprised the corresponding protein active site. To map the active site residues to as many Cp protein domains as possible, each PDB_ID used as a template for the homology modelling in CSA was assigned to the modelled ORFs.

As an alternative approach to determine the relevance of a given pocket (or residues), we looked for residues of a given Pfam family/domain that were located in an important position and are well conserved. Important positions were defined as those positions in the corresponding HMMer model whose information content was larger than a defined importance cut-off value (icov). The nature of the conserved amino acids in the corresponding position was determined by comparing each residue type emission probability (ep) with icov. If the ratio between ep and icov was larger than a conserved type cut-off value (ctcov), the corresponding residue type was assumed as conserved. Optimal values of icov and ctcov [11] were 0.27 and 0.24, respectively. These values were calculated as the ones that allowed labelling all the important residues of the CSA database in their respective protein's domains. Briefly, this approach is a strategy to extend the definition of catalytic site capturing all the sites described in CSA. CSA is a curated database with limited amount of data. Our strategy gives us a clue about new candidates of being catalytic sites, with some false positives.

By using these analyses, for each Pfam domain, the pipeline provided a list of position-residue type relevant residues, which could thus be mapped to all Cp ORFs with assigned Pfam domain.

### Pathogenicity islands and pan-modelomics information

Information collected from other software pipelines is used in the present work to enhance the target selection process.

All the ORFs that belonged to a pathogenicity island (PAIS) were properly labelled with this indicator. The PAIS were previously computed and identified using the PIPS software [24] that has predicted 16 pathogenicity islands in *C. pseudotuberculosis*. PIPS predicts pathogenicity islands by taking into account some important features, i.e., flanking tRNA, codon usage bias, GC content deviation; transposases, virulence factors and their absence in non-pathogenic organism of the same genus or related species. Pathogenicity islands are large regions that were acquired through horizontal gene transfer that represent the genome plasticity of a species and possess a high concentration of virulence factors. Virulence factors are proteins whose function is related to bacterial virulence and pathogenicity. They help the pathogenic bacterium in adhesion mechanisms, invasion, and surviving through colonization, and replication inside the host as well as in immune system evasion [24].

A novel integrative approach has been adapted in a recent work for the identification of new therapeutic targets in *C. pseudotuberculosis *[38] where a final set of 10 proteins has been selected that was essential for the bacterial survival. Here, too, a focus was made only on the previously selected homologous target proteins in the reference Cp1002 genome, whose detailed analysis with our pipeline is reported.

### Bacterial essential and non-host homologous proteins analysis

The pool of global highly druggable (GD) 58 target proteins was subjected to NCBI-BLASTp (*e-value *lesser than 1e-07, bit score ≥100, identity ≥ 50%) against human and ovine proteomes to identify non-host homologs targets, and coverage of all the sequences of the proteins greater than 80% [25]. The exhaustive list of GD and Virulent highly druggable (VD) ORFs is available in supplementary material **table S1**, additional file 1.

Furthermore, from the filtered list of 41 highly druggable non-host homologous target proteins, an approach based on a subtractive genomics was made for the conserved GD targets that were essential to bacteria [26]. Briefly, the set of proteins from *C. pseudotuberculosis *were submitted to the Database of Essential Genes (DEG, which contains experimentally validated essential genes from 20 bacteria) for homology analyses [27]. The BLASTp cut-off values used were: *e-value *= 1e^-05^, *bit score *≥100, *identity *≥ 35% [26]. The final list of targets based on previously described criteria contained 31 essential and non-host homologous target proteins. The list of putative targets was subjected to biochemical pathway analysis to KEGG (Kyoto Encyclopaedia of Genes and Genomes) [28], virulence using PIPS (Pathogenicity Island Prediction Software) [29], functionality using UniProt (Universal Protein Resource) [30], and cellular localization using CELLO (subCELlular LOcalization predictor) [31].

## Results and discussion

### Models in the reference Cp1002 strain

The following subsections describe the database data summary after running the HMMer software and the modelling pipeline.

A total of 2598 models were built from the ORFs of the reference genome and then a part of the ORFs were assigned to the Pfam families. Here, 1206 unique ORFs were involved. The models from the Pfam family were 1546 in total, where only 879 unique Pfam families were used. The other 1051 models correspond to full ORF models (table 1).

### Models in non-reference strains

The details of these constructed models are presented here in a tabulated form.

**Table 1:** Summary of modelling pipeline over non-reference strains.

NCBI accession	*C. pseudotuberculosis *strains	ORFs	Homolog ORFs to Cp1002	Modelled ORFs
NC_017308.1	Cp1/06-A	1963	1654	493

NC_017730.1	Cp31	2088	1663	441

NC_017945.1	Cp258	2106	1661	503

NC_016932.1	Cp316	2057	1696	502

NC_017307.1	CpCIP52.97	2057	1704	489

NC_018019.1	Cp162	2002	1652	444

NC_017031.1	CpP54B96	2084	1808	543

NC_017462.1	Cp267	2148	1826	561

NC_017306.1	Cp42/02-A	2097	1783	544

NC_017301.1	CpC231	2051	1821	552

NC_017303.1	CpI19	2095	1826	552

NC_016781.1	Cp3/99-5	2099	1759	530

NC_017305.1	CpPAT10	2142	1814	542

NC_014329.1	CpFRC41	2089	1827	559

### Druggability summary of 15 *C. pseudotuberculosis *strains

A summary of the calculated structural druggability scores is presented in table 2. In parentheses are the pockets containing residues from CSA database or at least one important residue from the Pfam family, assigned to the corresponding ORF in the Cp1002 genome. It should be noted that the pocket calculations over the non-reference strains were done only for the homologous models (proteins with identity > 85% to the reference genome). ORFs with high druggability over all the strains are labelled as Global druggable and those druggable in the pathogenic islands as Virulent druggable. For Cp1002 strain, only those ORFs in the list appear having at least one homolog ORF in another strain. In other strains, only the ORFs homologous to a Cp1002 ORF are considered for this table.

**Table 2:** Summary of druggability for all *C. pseudotuberculosis *strains, Global highly druggable (GD) ORFs and Virulent highly druggable (VD) ORFs.

NCBI accession	*C. pseudotuberculosis *Strain	ND or PD	D	HD
NC_017300.1	Cp1002	20	185	377

NC_017308.1	Cp1/06-A	42	224	227

NC_017730.1	Cp31	36	202	203

NC_017945.1	Cp258	39	226	238

NC_016932.1	Cp316	44	232	226

NC_017307.1	CpCIP52.97	39	227	223

NC_018019.1	Cp162	32	196	216

NC_017031.1	CpP54B96	43	241	259

NC_017462.1	Cp267	44	255	262

NC_017306.1	Cp42/02-A	45	249	250

NC_017301.1	CpC231	48	248	256

NC_017303.1	CpI19	44	251	257

NC_016781.1	Cp3/99-5	44	246	240

NC_017305.1	CpPAT10	42	246	254

NC_014329.1	CpFRC41	46	251	262

	**Global highly druggable (GD) ORFs**			58

	**Virulent highly druggable (VD) ORFs**			2

### Bacterial essential and non-host homologous proteins analyses

#### An exhaustive literature review of candidate druggable targets

As aforementioned, the list of 58 most druggable proteins (**table S1**, additional file 1) was compared to the corresponding host proteomes, leading to the identification of 41 non-host homologous proteins (**table S2**, additional file 1) and 17 host homologous proteins (**table S3**, additional file 1). A final list of 31 essential and non-host homologous targets from *C. pseudotuberculosis *is given in **table S4**, additional file 1 after computing the 41 non-host homologous proteins to the database of essential genes (DEG), using the earlier mentioned default parameters.

Based on this filter, the number of selected targets was drastically reduced to a final set of 31 targets. This list was considered as druggable, essential and non-host homologous target proteins. We have further extrapolated our search to find out whether some of these putative targets have already been reported in the literature or not. This way 9 such putative targets were found where 3 of them including 3-isopropylmalate dehydratase small subunit, 50S ribosomal protein L30 and Chromosomal replication initiator protein DnaA have previously been reported in *C. pseudotuberculosis *[25,26], while the other 6 druggable target proteins have been reported in other pathogenic microorganisms, both in bacteria and parasites (table 3). The remaining 22 druggable targets that are not yet reported as putative targets were searched for molecular functions, biological processes, cellular compartmentalisations and metabolic pathway roles (**table S5**, additional file 1).

**Table 3:** Essential and non-host homologous bacterial protein already reported as drug target in other pathogenic microorganisms.

Sr. No	Reference Protein Loci	UniProt Accession	Protein Name	PMID/Reference
1	Cp1002_0854	D9Q7U7	1,4-alpha-glucan branching enzyme GlgB	[32]

2	Cp1002_0907	D9Q7Z8	3-isopropylmalate dehydratase small subunit	[25]

3	Cp1002_0948	D9Q838	Acetylglutamate kinase	[33]

4	Cp1002_0412	D9Q6L7	Alanine racemase	[34]

5	Cp1002_0374	D9Q6I0	50S ribosomal protein L30	[26]

6	Cp1002_1592	D9Q409	Bifunctional protein folC	[35]

7	Cp1002_1239	D9Q8X6	Diaminopimelate epimerase	[36]

8	Cp1002_1270	D9Q906	Riboflavin biosynthesis protein ribF	[37]

9	Cp1002_0001	D9Q5G4	Chromosomal replication initiator protein DnaA	[25]

## Conclusions and perspectives

In the present work we have attempted to show a comprehensive study of the druggability scores along the known completely sequenced strains of *C. pseudotuberculosis *species to complement further the research work performed by our colleagues and collaborators. After our pipeline was executed, a list of cross-strain highly druggable 58 ORFs was obtained. These ORFs had the information about their membership in the set of virulent strains. We too have provided the information if the most druggable pockets of the ORF have highly conserved residues in the Pfam families they belong to, and if a catalytic site is reported in the template structure or structures that were used to build the homology model for each ORF. We expect that the constructed dataset might serve as a guide for the scientific community to have a better understanding while selecting protein candidates as therapeutic and druggable ones. All the data is available via web at http://www.inquimae.fcen.uba.ar/turjanski_adrian.

The information obtained here was also used to exhaustively analyse the target proteins actually considered as druggable targets in previous works. Our analyses have proposed a set of putative druggable targets in the veterinary pathogen *C. pseudotuberculosis *on one hand, while on the other hand it has also demonstrated the efficiency and the high-throughput nature of our pipeline used in this study. All this work is expandable and could be applied to the emergence of new strains of the same organism species as well as to the new organisms with the same characteristics.

## Competing interests

The authors declare that they have no competing interests.

## Authors' contributions

Coordinated entire work: LGR SSH EL AGT. Performed all *in silico *analyses: LGR SSH EL ST SBJ. Cross-analysed genome contents, conserved pan-modelome, subtractive/genome modelome approach, residue level structural comparison: SSH LGR EL DB AGT. Provided timely consultation and reviewed the manuscript: VA DB AA JA RSF AS. Read and approved the final manuscript: RSF SSH JA DB AGT AS VA. Conceived and designed the work: SSH LGR EL. Analysed the data: SSH RSF ST SBJ DB AGT AM AS VA. Wrote the paper: LGR SSH AGT.

## Supplementary Material

Additional file 1 **Supplementary tables**. Click here for file

## Declarations

Publication costs for this article were funded by grant from the Argentinian National Agency PICT-2010-2805 and VA from the Programa Nacional de Pós-doutorado da CAPES-PNPD/2011: Concessão Institucional, Brazil.

This article has been published as part of *BMC Genomics *Volume 16 Supplement 5, 2015: Proceedings of the 10th International Conference of the Brazilian Association for Bioinformatics and Computational Biology (X-Meeting 2014). The full contents of the supplement are available online at http://www.biomedcentral.com/bmcgenomics/supplements/16/S5.
