# Supplementary material for: An integrated structural proteomics approach along the druggable genome of Corynebacterium pseudotuberculosis species for putative druggable targets
Source: BMC Genomics. 2015 May 26;16(Suppl 5):S9. doi: 10.1186/1471-2164-16-S5-S9 (PMC4460585; doi:10.1186/1471-2164-16-S5-S9)
Supplement: Additional file 1 — Supplementary tables. [file 1471-2164-16-S5-S9-S1.doc]

# **Additional files**

**Table S1, additional file 1**: **The 58 proteins with high druggability values and the families detected for ORFs in the reference *C. pseudotuberculosis* strain (Cp1002).**

The druggability in the reference genome is listed, for other strains, to remain with high druggability values, they need to have a value > 0.8. ORFs that belong to PAIS of the reference genome are also highlighted. Those ORFs are also highlighted whose most druggable pockets are in contact with the important AAs (amino acids) labelled via CSA or Pfam databases. Some proteins have modelled domains that are only a part of the whole protein, the pertinence of such pockets founded are subjected to further analysis. Off-targeting information is given for host organisms including Homo sapiens. This information allows discarding some of the global druggable targets.

| **S.No** | **Reference Protein Loci** | **UniProt ID** | **Protein Name** | **Protein Family** | **Druggabilty in Cp1002** | **PAIS** | **Essential for Pathogen** | **Host Homology** |
| --- | --- | --- | --- | --- | --- | --- | --- | --- |
| 1 | Cp1002_0854 | D9Q7U7 | 1,4-alpha-glucan branching enzyme GlgB | PF00128 | 0.983 | No | Yes | No |
| 2 | Cp1002_0543 | D9Q6Z2 | Trk system potassium uptake protein trkA | PF02254 | 0.978 | No | No | Yes |
| 3 | Cp1002_1369 | D9Q3E4 | Ribosome-associated heat shock protein/S4 | PF01479 | 0.974 | No | No | No |
| 4 | Cp1002_1382 | D9Q3F7 | Isoleucine--tRNA ligase | PF08264 | 0.969 | No | No | Yes |
| 5 | Cp1002_1381 | D9Q3Z8 | Transport YidE/YbjL family protein | PF02080 | 0.966 | No | Yes | No |
| 6 | Cp1002_0907 | D9Q7Z8 | 3-isopropylmalate dehydratase small subunit | PF00694 | 0.957 | No | Yes | No |
| 7 | Cp1002_1795 | D9Q4K8 | Hypoxanthine-guanine phosphoribosyltransferase | PF00156 | 0.952 | No | Yes | No |
| 8 | Cp1002_1327 | D9Q3A4 | Nitrogen regulatory protein P-II | PF00543 | 0.943 | No | No | Yes |
| 9 | Cp1002_1141 | D9Q8N1 | Chorismate synthase | PF01264 | 0.942 | No | No | Yes |
| 10 | Cp1002_1072 | D9Q8G2 | Ferrochelatase | PF00762 | 0.939 | No | Yes | No |
| 11 | Cp1002_1186 | D9Q8S5 | Threonine--tRNA ligase | PF03129 | 0.938 | No | Yes | No |
| 12 | Cp1002_0370 | D9Q6H6 | 30S ribosomal protein S8 | PF00410 | 0.934 | No | Yes | No |
| 13 | Cp1002_1189 | D9Q8S8 | Copper resistance protein CopC | PF04234 | 0.929 | No | No | No |
| 14 | Cp1002_0948 | D9Q838 | Acetylglutamate kinase | PF00696 | 0.924 | No | Yes | No |
| 15 | Cp1002_0311 | D9Q6B8 | Aminopeptidase C | PF03051 | 0.922 | No | No | No |
| 16 | Cp1002_0861 | D9Q7V4 | Cysteine desulfurase | PF00266 | 0.922 | No | Yes | No |
| 17 | Cp1002_0676 | D9Q7C4 | Glycine betaine transporter | PF02028 | 0.919 | No | No | No |
| 18 | Cp1002_0228 | D9Q636 | DNA polymerase III subunit gamma/tau | PF13177 | 0.914 | No | Yes | No |
| 19 | Cp1002_1213 | D9Q8V1 | Diphtheria toxin repressor | PF02742 | 0.911 | No | Yes | No |
| 20 | Cp1002_0782 | D9Q7M7 | Glycosyl transferase group 2 | PF00535 | 0.907 | No | No | Yes |
| 21 | Cp1002_0412 | D9Q6L7 | Alanine racemase | PF00842 | 0.906 | No | Yes | No |
| 22 | Cp1002_1148 | D9Q8N7 | Uncharacterized AAA domain-containing protein Rv2559c/MT2636 | PF00004 | 0.905 | No | No | Yes |
| 23 | Cp1002_0198 | D9Q606 | Uncharacterized metallophosphoesterase ykuE | PF12850 | 0.905 | No | No | Yes |
| 24 | Cp1002_0728 | D9Q7H4 | Serine hydroxymethyltransferase | PF00464 | 0.903 | No | No | Yes |
| 25 | Cp1002_1338 | D9Q3B3 | Alanine dehydrogenase | PF01262 | 0.899 | No | No | No |
| 26 | Cp1002_0258 | D9Q666 | 2,3-bisphosphoglycerate-dependent phosphoglycerate mutase | PF00300 | 0.899 | No | No | Yes |
| **27** | **Cp1002_0042** | **D9Q5K5** | **Transcription regulator padR** | **PF03551** | **0.894** | **Yes** | **No** | **Yes** |
| 28 | Cp1002_2084 | D9Q5D7 | Anthranilate synthase component II | PF00117 | 0.893 | No | Yes | No |
| 29 | Cp1002_0436 | D9Q6N9 | D-methionine-binding lipoprotein metQ | PF03180 | 0.881 | No | Yes | No |
| 30 | Cp1002_0259 | D9Q667 | Signal-transduction histidine kinase senX3 | PF02518 | 0.88 | No | Yes | No |
| 31 | Cp1002_0374 | D9Q6I0 | 50S ribosomal protein L30 | PF00327 | 0.88 | No | Yes | No |
| 32 | Cp1002_1592 | D9Q409 | Bifunctional protein folC | PF02875 | 0.877 | No | Yes | No |
| 33 | Cp1002_1239 | D9Q8X6 | Diaminopimelate epimerase | PF01678 | 0.876 | No | Yes | No |
| 34 | Cp1002_0629 | D9Q777 | Precorrin-6A synthase (Deacetylating) | PF00590 | 0.873 | No | No | No |
| 35 | Cp1002_1270 | D9Q906 | Riboflavin biosynthesis protein ribF | PF06574 | 0.87 | No | Yes | No |
| 36 | Cp1002_1338 | D9Q3B3 | Alanine dehydrogenase | PF05222 | 0.869 | No | No | No |
| 37 | Cp1002_1350 | D9Q3C5 | Phosphoribosyl-AMP cyclohydrolase | PF01502 | 0.862 | No | No | Yes |
| 38 | Cp1002_1363 | D9Q3D8 | TetR family transcriptional regulator | PF00440 | 0.857 | No | No | No |
| 39 | Cp1002_1982 | D9Q537 | LytR family transcriptional regulator | PF03816 | 0.854 | No | Yes | No |
| 40 | Cp1002_1051 | D9Q8E1 | Aldo/keto reductase family oxidoreductase | PF00248 | 0.852 | No | No | Yes |
| 41 | Cp1002_0440 | D9Q6P3 | Manganese ABC transporter ATP-binding protein | PF00005 | 0.852 | No | No | Yes |
| 42 | Cp1002_0846 | D9Q7T9 | ATP synthase gamma chain | PF00231 | 0.848 | No | Yes | No |
| 43 | Cp1002_0679 | D9Q7C7 | Methionine--tRNA ligase | PF09334 | 0.845 | No | Yes | No |
| 44 | Cp1002_0917 | D9Q808 | Ribosomal RNA small subunit methyltransferase D | PF03602 | 0.843 | No | Yes | No |
| 45 | Cp1002_1697 | D9Q4B2 | Transcriptional regulator, LuxR family | PF00196 | 0.841 | No | No | No |
| 46 | Cp1002_0035 | D9Q5J8 | Penicillin-binding protein A | PF00905 | 0.834 | No | No | Yes |
| 47 | Cp1002_1800 | D9Q4L3 | Aerobic C4-dicarboxylate transport protein | PF00375 | 0.832 | No | Yes | No |
| 48 | Cp1002_1994 | D9Q548 | Flavin reductase | PF03358 | 0.83 | No | No | No |
| 49 | Cp1002_1797 | D9Q4L0 | D-alanyl-D-alanine carboxypeptidase | PF02113 | 0.828 | No | No | Yes |
| 50 | Cp1002_0199 | D9Q607 | Uncharacterized protein yqeY | PF09424 | 0.828 | No | Yes | No |
| 51 | Cp1002_0001 | D9Q5G4 | Chromosomal replication initiator protein DnaA | PF00308 | 0.825 | No | Yes | No |
| 52 | Cp1002_2094 | D9Q5E7 | Uncharacterized protein | PF02622 | 0.825 | No | Yes | No |
| **53** | **Cp1002_0139** | **D9Q5V0** | **Zinc-binding alcohol dehydrogenase** | **PF08240** | **0.822** | **Yes** | **Yes** | **No** |
| 54 | Cp1002_0775 | D9Q7M0 | Aromatic amino acid transport protein | PF00324 | 0.821 | No | Yes | No |
| 55 | Cp1002_1670 | D9Q486 | Cytochrome C oxidase polypeptide I | PF00115 | 0.816 | No | Yes | No |
| 56 | Cp1002_1276 | D9Q912 | Ribosome-binding factor A | PF02033 | 0.81 | No | Yes | No |
| 57 | Cp1002_1028 | D9Q8B8 | ATP-dependent DNA helicase | PF00270 | 0.809 | No | No | Yes |
| 58 | Cp1002_1037 | D9Q8C7 | SAM-dependent methyltransferase involved in tRNA-Met maturation | PF08704 | 0.803 | No | No | Yes |

**Table S2, additional file 2**: **Globally Druggable 41 targets that don’t match with any protein on *Homo sapiens* or other host organisms.**

| **Sr. No** | **Reference Protein Loci** | **UniProt Accession** | **Protein Name** | **Druggabilty in Cp1002** |
| --- | --- | --- | --- | --- |
| 1 | Cp1002_0854 | D9Q7U7 | 1,4-alpha-glucan branching enzyme GlgB | 0.983 |
| 2 | Cp1002_1381 | D9Q3Z8 | Transport YidE/YbjL family protein | 0.966 |
| 3 | Cp1002_0907 | D9Q7Z8 | 3-isopropylmalate dehydratase small subunit | 0.957 |
| 4 | Cp1002_1795 | D9Q4K8 | Hypoxanthine-guanine phosphoribosyltransferase | 0.952 |
| 5 | Cp1002_1072 | D9Q8G2 | Ferrochelatase | 0.939 |
| 6 | Cp1002_1186 | D9Q8S5 | Threonine--tRNA ligase | 0.938 |
| 7 | Cp1002_0370 | D9Q6H6 | 30S ribosomal protein S8 | 0.934 |
| 8 | Cp1002_1189 | D9Q8S8 | Copper resistance protein CopC | 0.929 |
| 9 | Cp1002_0948 | D9Q838 | Acetylglutamate kinase | 0.924 |
| 10 | Cp1002_0311 | D9Q6B8 | Aminopeptidase C | 0.922 |
| 11 | Cp1002_0861 | D9Q7V4 | Cysteine desulfurase | 0.922 |
| 12 | Cp1002_0676 | D9Q7C4 | Glycine betaine transporter | 0.919 |
| 13 | Cp1002_0228 | D9Q636 | DNA polymerase III subunit gamma/tau | 0.914 |
| 14 | Cp1002_1213 | D9Q8V1 | Diphtheria toxin repressor | 0.911 |
| 15 | Cp1002_0412 | D9Q6L7 | Alanine racemase | 0.906 |
| 16 | Cp1002_1338 | D9Q3B3 | Alanine dehydrogenase | 0.899 |
| 17 | Cp1002_2084 | D9Q5D7 | Anthranilate synthase component II | 0.893 |
| 18 | Cp1002_0436 | D9Q6N9 | D-methionine-binding lipoprotein metQ | 0.881 |
| 19 | Cp1002_0259 | D9Q667 | Signal-transduction histidine kinase senX3 | 0.88 |
| 20 | Cp1002_0374 | D9Q6I0 | 50S ribosomal protein L30 | 0.88 |
| 21 | Cp1002_1592 | D9Q409 | Bifunctional protein folC | 0.877 |
| 22 | Cp1002_1239 | D9Q8X6 | Diaminopimelate epimerase | 0.876 |
| 23 | Cp1002_0629 | D9Q777 | Precorrin-6A synthase (Deacetylating) | 0.873 |
| 24 | Cp1002_1270 | D9Q906 | Riboflavin biosynthesis protein ribF | 0.87 |
| 25 | Cp1002_1338 | D9Q3B3 | Alanine dehydrogenase | 0.869 |
| 26 | Cp1002_1363 | D9Q3D8 | TetR family transcriptional regulator | 0.857 |
| 27 | Cp1002_1982 | D9Q537 | LytR family transcriptional regulator | 0.854 |
| 28 | Cp1002_0846 | D9Q7T9 | ATP synthase gamma chain | 0.848 |
| 29 | Cp1002_0679 | D9Q7C7 | Methionine--tRNA ligase | 0.845 |
| 30 | Cp1002_0917 | D9Q808 | Ribosomal RNA small subunit methyltransferase D | 0.843 |
| 31 | Cp1002_1697 | D9Q4B2 | Transcriptional regulator, LuxR family | 0.841 |
| 32 | Cp1002_1800 | D9Q4L3 | Aerobic C4-dicarboxylate transport protein | 0.832 |
| 33 | Cp1002_0001 | D9Q5G4 | Chromosomal replication initiator protein DnaA | 0.825 |
| 34 | Cp1002_2094 | D9Q5E7 | Uncharacterized protein | 0.825 |
| 35 | Cp1002_0775 | D9Q7M0 | Aromatic amino acid transport protein | 0.821 |
| 36 | Cp1002_1670 | D9Q486 | Cytochrome C oxidase polypeptide I | 0.816 |
| 37 | Cp1002_1276 | D9Q912 | Ribosome-binding factor A | 0.81 |
| 38 | Cp1002_1369 | D9Q3E4 | Ribosome-associated heat shock protein/S4 | 0.974 |
| 39 | Cp1002_0139 | D9Q5V0 | Zinc-binding alcohol dehydrogenase | 0.822 |
| 40 | Cp1002_0199 | D9Q607 | Uncharacterized protein yqeY | 0.828 |
| 41 | Cp1002_1994 | D9Q548 | Flavin reductase | 0.83 |

**Table S3, additional file 3**: List of 17 host homologous proteins with all cut-off and identity values to corresponding hosts.

| **Sr. No** | **Reference Protein Loci** | **UniProt Accession** | **Protein Name** | **Host: UniProt Accession**  **Cut-off Values** |
| --- | --- | --- | --- | --- |
| 1 | Cp1002_0543 | D9Q6Z2 | Trk system potassium uptake protein trkA | Homo Sapiens:  E9PJX2, identity=56%, coverage=96% |
| 2 | Cp1002_1382 | D9Q3F7 | Isoleucine--tRNA ligase | Sheep:  W5PLQ3, identity=70,coverage=94% |
| 3 | Cp1002_1327 | D9Q3A4 | Nitrogen regulatory protein P-II | Sheep:  W5PAY0, identity=58%, coverage=95% |
| 4 | Cp1002_1141 | D9Q8N1 | Chorismate synthase | Goat:  Q8HY38, identity =55% coverage=94% |
| 5 | Cp1002_1148 | D9Q8N7 | Uncharacterized AAA domain-containing protein Rv2559c/MT2636 | Homo Sapiens:  H0YAL2, identity =66% coverage =93%  Sheep:  W5Q3H5, identity =66% coverage =93% |
| 6 | Cp1002_0198 | D9Q606 | Uncharacterized metallophosphoesterase ykuE | Goat:  G1DFW1, identity =56% coverage =93% |
| 7 | Cp1002_0728 | D9Q7H4 | Serine hydroxymethyltransferase | Homo Sapiens:  J3KRK5 identity =55% coverage =58% |
| 8 | Cp1002_0258 | D9Q666 | 2,3-bisphosphoglycerate-dependent phosphoglycerate mutase | Homo Sapiens:  P18669, identity =52% coverage =97%  Sheep:  W5PVY5, identity =50% coverage =97% |
| 9 | Cp1002_0042 | D9Q5K5 | Transcription regulator padR | Sheep:  W5NSE4, identity =51% coverage =96% |
| 10 | Cp1002_1350 | D9Q3C5 | Phosphoribosyl-AMP cyclohydrolase | Sheep:  W5P1Z5, identity =64% coverage =92% |
| 11 | Cp1002_1051 | D9Q8E1 | Aldo/keto reductase family oxidoreductase | Goat:  G1DGE6, identity =52% coverage =94% |
| 12 | Cp1002_0440 | D9Q6P3 | Manganese ABC transporter ATP-binding protein | Homo Sapiens:  Q9NUQ8, identity =66% coverage =93%Sheep:  W5Q081, identity =71% coverage =92% |
| 13 | Cp1002_0035 | D9Q5J8 | Penicillin-binding protein A | Homo Sapiens:  A8MUL3, identity =53% coverage =93% |
| 14 | Cp1002_1797 | D9Q4L0 | D-alanyl-D-alanine carboxypeptidase | Sheep:  W5PSW2, identity =62% coverage =93% |
| 15 | Cp1002_1028 | D9Q8B8 | ATP-dependent DNA helicase | Homo Sapiens:  Q6ZMI0, identity =51% coverage =96%  Goat:  O18809, identity =52% coverage =94% |
| 16 | Cp1002_1037 | D9Q8C7 | SAM-dependent methyltransferase involved in tRNA-Met maturation | Goat:  Q68G89, identity =52% coverage =93% |
| 17 | Cp1002_0782 | D9Q7M7 | Glycosyl transferase group 2 | Sheep:  W5Q161, identity =53% coverage =93% |

**Table S4, additional file 4**: Final list of 31 non-host homologous targets computed to DEG database, the essential proteins.

| **S.No** | **Reference Protein Loci** | **Protein Name** | **Hits with DEG database protein** |
| --- | --- | --- | --- |
| 1 | **Cp1002_0854^#^** | 1,4-alpha-glucan branching enzyme GlgB | [DEG10100210;](http://tubic.tju.edu.cn/deg/information.php?ac=DEG10100210&db=p)[DEG10050489;](http://tubic.tju.edu.cn/deg/information.php?ac=DEG10050489&db=p)[DEG10110090;](http://tubic.tju.edu.cn/deg/information.php?ac=DEG10110090&db=p)[DEG10250247;](http://tubic.tju.edu.cn/deg/information.php?ac=DEG10250247&db=p)[DEG10250311;](http://tubic.tju.edu.cn/deg/information.php?ac=DEG10250311&db=p)[DEG10270293;](http://tubic.tju.edu.cn/deg/information.php?ac=DEG10270293&db=p)[DEG10270295;](http://tubic.tju.edu.cn/deg/information.php?ac=DEG10270295&db=p)[DEG10270241;](http://tubic.tju.edu.cn/deg/information.php?ac=DEG10270241&db=p) |
| 2 | Cp1002_1381 | Transport YidE/YbjL family protein | [DEG10050015;](http://tubic.tju.edu.cn/deg/information.php?ac=DEG10050015&db=p) |
| 3 | **Cp1002_0907^#^** | 3-isopropylmalate dehydratase small subunit | [DEG10270535;](http://tubic.tju.edu.cn/deg/information.php?ac=DEG10270535&db=p)[DEG10100478;](http://tubic.tju.edu.cn/deg/information.php?ac=DEG10100478&db=p)[DEG10290373;](http://tubic.tju.edu.cn/deg/information.php?ac=DEG10290373&db=p)[DEG10280513;](http://tubic.tju.edu.cn/deg/information.php?ac=DEG10280513&db=p)[DEG10250583;](http://tubic.tju.edu.cn/deg/information.php?ac=DEG10250583&db=p)[DEG10050349;](http://tubic.tju.edu.cn/deg/information.php?ac=DEG10050349&db=p)[DEG10130079;](http://tubic.tju.edu.cn/deg/information.php?ac=DEG10130079&db=p) |
| 4 | Cp1002_1795 | Hypoxanthine-guanine phosphoribosyltransferase | [DEG10140209;](http://tubic.tju.edu.cn/deg/information.php?ac=DEG10140209&db=p)[DEG10220168;](http://tubic.tju.edu.cn/deg/information.php?ac=DEG10220168&db=p)[DEG10060370;](http://tubic.tju.edu.cn/deg/information.php?ac=DEG10060370&db=p)[DEG10070003;](http://tubic.tju.edu.cn/deg/information.php?ac=DEG10070003&db=p)[DEG10340157;](http://tubic.tju.edu.cn/deg/information.php?ac=DEG10340157&db=p)[DEG10010017;](http://tubic.tju.edu.cn/deg/information.php?ac=DEG10010017&db=p)[DEG10270636;](http://tubic.tju.edu.cn/deg/information.php?ac=DEG10270636&db=p)[DEG10250705;](http://tubic.tju.edu.cn/deg/information.php?ac=DEG10250705&db=p) |
| 5 | Cp1002_1072 | Ferrochelatase | [DEG10270277;](http://tubic.tju.edu.cn/deg/information.php?ac=DEG10270277&db=p)[DEG10250294;](http://tubic.tju.edu.cn/deg/information.php?ac=DEG10250294&db=p)[DEG10100252;](http://tubic.tju.edu.cn/deg/information.php?ac=DEG10100252&db=p)[DEG10240155;](http://tubic.tju.edu.cn/deg/information.php?ac=DEG10240155&db=p) |
| 6 | Cp1002_1186 | Threonine--tRNA ligase | [DEG10030594;](http://tubic.tju.edu.cn/deg/information.php?ac=DEG10030594&db=p)[DEG10060308;](http://tubic.tju.edu.cn/deg/information.php?ac=DEG10060308&db=p)[DEG10120270;](http://tubic.tju.edu.cn/deg/information.php?ac=DEG10120270&db=p)[DEG10220196;](http://tubic.tju.edu.cn/deg/information.php?ac=DEG10220196&db=p)[DEG10100421;](http://tubic.tju.edu.cn/deg/information.php?ac=DEG10100421&db=p)[DEG10080016;](http://tubic.tju.edu.cn/deg/information.php?ac=DEG10080016&db=p)[DEG10340211;](http://tubic.tju.edu.cn/deg/information.php?ac=DEG10340211&db=p)[DEG10130387;](http://tubic.tju.edu.cn/deg/information.php?ac=DEG10130387&db=p)[DEG10250516;](http://tubic.tju.edu.cn/deg/information.php?ac=DEG10250516&db=p)[DEG10210142;](http://tubic.tju.edu.cn/deg/information.php?ac=DEG10210142&db=p)[DEG10110078;](http://tubic.tju.edu.cn/deg/information.php?ac=DEG10110078&db=p)[DEG10050493;](http://tubic.tju.edu.cn/deg/information.php?ac=DEG10050493&db=p)[DEG10170248;](http://tubic.tju.edu.cn/deg/information.php?ac=DEG10170248&db=p)[DEG10290210;](http://tubic.tju.edu.cn/deg/information.php?ac=DEG10290210&db=p)[DEG10160093;](http://tubic.tju.edu.cn/deg/information.php?ac=DEG10160093&db=p)[DEG10200074;](http://tubic.tju.edu.cn/deg/information.php?ac=DEG10200074&db=p)[DEG10230015;](http://tubic.tju.edu.cn/deg/information.php?ac=DEG10230015&db=p)[DEG10140296;](http://tubic.tju.edu.cn/deg/information.php?ac=DEG10140296&db=p)[DEG10020192;](http://tubic.tju.edu.cn/deg/information.php?ac=DEG10020192&db=p)[DEG10190121;](http://tubic.tju.edu.cn/deg/information.php?ac=DEG10190121&db=p)[DEG10070202;](http://tubic.tju.edu.cn/deg/information.php?ac=DEG10070202&db=p)[DEG10270481;](http://tubic.tju.edu.cn/deg/information.php?ac=DEG10270481&db=p)[DEG10330095;](http://tubic.tju.edu.cn/deg/information.php?ac=DEG10330095&db=p)[DEG10180289;](http://tubic.tju.edu.cn/deg/information.php?ac=DEG10180289&db=p)[DEG10320127;](http://tubic.tju.edu.cn/deg/information.php?ac=DEG10320127&db=p) |
| 7 | Cp1002_0370 | 30S ribosomal protein S8 | [DEG10240325;](http://tubic.tju.edu.cn/deg/information.php?ac=DEG10240325&db=p)[DEG10130418;](http://tubic.tju.edu.cn/deg/information.php?ac=DEG10130418&db=p)[DEG10180497;](http://tubic.tju.edu.cn/deg/information.php?ac=DEG10180497&db=p)[DEG10120067;](http://tubic.tju.edu.cn/deg/information.php?ac=DEG10120067&db=p)[DEG10050279;](http://tubic.tju.edu.cn/deg/information.php?ac=DEG10050279&db=p)[DEG10020264;](http://tubic.tju.edu.cn/deg/information.php?ac=DEG10020264&db=p)[DEG10290046;](http://tubic.tju.edu.cn/deg/information.php?ac=DEG10290046&db=p)[DEG10250142;](http://tubic.tju.edu.cn/deg/information.php?ac=DEG10250142&db=p)[DEG10220407;](http://tubic.tju.edu.cn/deg/information.php?ac=DEG10220407&db=p)[DEG10210021;](http://tubic.tju.edu.cn/deg/information.php?ac=DEG10210021&db=p)[DEG10270130;](http://tubic.tju.edu.cn/deg/information.php?ac=DEG10270130&db=p)[DEG10140227;](http://tubic.tju.edu.cn/deg/information.php?ac=DEG10140227&db=p)[DEG10280169;](http://tubic.tju.edu.cn/deg/information.php?ac=DEG10280169&db=p)[DEG10320275;](http://tubic.tju.edu.cn/deg/information.php?ac=DEG10320275&db=p)[DEG10100116;](http://tubic.tju.edu.cn/deg/information.php?ac=DEG10100116&db=p)[DEG10200149;](http://tubic.tju.edu.cn/deg/information.php?ac=DEG10200149&db=p)[DEG10010049;](http://tubic.tju.edu.cn/deg/information.php?ac=DEG10010049&db=p)[DEG10170320;](http://tubic.tju.edu.cn/deg/information.php?ac=DEG10170320&db=p)[DEG10060136;](http://tubic.tju.edu.cn/deg/information.php?ac=DEG10060136&db=p)[DEG10340467;](http://tubic.tju.edu.cn/deg/information.php?ac=DEG10340467&db=p)[DEG10310217;](http://tubic.tju.edu.cn/deg/information.php?ac=DEG10310217&db=p)[DEG10030523;](http://tubic.tju.edu.cn/deg/information.php?ac=DEG10030523&db=p)[DEG10160314;](http://tubic.tju.edu.cn/deg/information.php?ac=DEG10160314&db=p)[DEG10190215;](http://tubic.tju.edu.cn/deg/information.php?ac=DEG10190215&db=p)[DEG10330318;](http://tubic.tju.edu.cn/deg/information.php?ac=DEG10330318&db=p) |
| 8 | **Cp1002_0948^#^** | Acetylglutamate kinase | [DEG10280313;](http://tubic.tju.edu.cn/deg/information.php?ac=DEG10280313&db=p)[DEG10130151;](http://tubic.tju.edu.cn/deg/information.php?ac=DEG10130151&db=p)[DEG10100282;](http://tubic.tju.edu.cn/deg/information.php?ac=DEG10100282&db=p) |
| 9 | Cp1002_0861 | Cysteine desulfurase | [DEG10340054;](http://tubic.tju.edu.cn/deg/information.php?ac=DEG10340054&db=p)[DEG10030158;](http://tubic.tju.edu.cn/deg/information.php?ac=DEG10030158&db=p)[DEG10290203;](http://tubic.tju.edu.cn/deg/information.php?ac=DEG10290203&db=p)[DEG10250595;](http://tubic.tju.edu.cn/deg/information.php?ac=DEG10250595&db=p)[DEG10050462;](http://tubic.tju.edu.cn/deg/information.php?ac=DEG10050462&db=p)[DEG10220155;](http://tubic.tju.edu.cn/deg/information.php?ac=DEG10220155&db=p)[DEG10020180;](http://tubic.tju.edu.cn/deg/information.php?ac=DEG10020180&db=p)[DEG10200232;](http://tubic.tju.edu.cn/deg/information.php?ac=DEG10200232&db=p)[DEG10020079;](http://tubic.tju.edu.cn/deg/information.php?ac=DEG10020079&db=p)[DEG10200236;](http://tubic.tju.edu.cn/deg/information.php?ac=DEG10200236&db=p)[DEG10240212;](http://tubic.tju.edu.cn/deg/information.php?ac=DEG10240212&db=p)[DEG10250285;](http://tubic.tju.edu.cn/deg/information.php?ac=DEG10250285&db=p)[DEG10100244;](http://tubic.tju.edu.cn/deg/information.php?ac=DEG10100244&db=p)[DEG10100490;](http://tubic.tju.edu.cn/deg/information.php?ac=DEG10100490&db=p)[DEG10210119;](http://tubic.tju.edu.cn/deg/information.php?ac=DEG10210119&db=p)[DEG10010234;](http://tubic.tju.edu.cn/deg/information.php?ac=DEG10010234&db=p)[DEG10170226;](http://tubic.tju.edu.cn/deg/information.php?ac=DEG10170226&db=p)[DEG10270547;](http://tubic.tju.edu.cn/deg/information.php?ac=DEG10270547&db=p)[DEG10280316;](http://tubic.tju.edu.cn/deg/information.php?ac=DEG10280316&db=p)[DEG10320201;](http://tubic.tju.edu.cn/deg/information.php?ac=DEG10320201&db=p)[DEG10110161;](http://tubic.tju.edu.cn/deg/information.php?ac=DEG10110161&db=p)[DEG10160058;](http://tubic.tju.edu.cn/deg/information.php?ac=DEG10160058&db=p)[DEG10230199;](http://tubic.tju.edu.cn/deg/information.php?ac=DEG10230199&db=p)[DEG10330059;](http://tubic.tju.edu.cn/deg/information.php?ac=DEG10330059&db=p)[DEG10070166;](http://tubic.tju.edu.cn/deg/information.php?ac=DEG10070166&db=p)[DEG10170086;](http://tubic.tju.edu.cn/deg/information.php?ac=DEG10170086&db=p)[DEG10130211;](http://tubic.tju.edu.cn/deg/information.php?ac=DEG10130211&db=p)[DEG10180388;](http://tubic.tju.edu.cn/deg/information.php?ac=DEG10180388&db=p)[DEG10010191;](http://tubic.tju.edu.cn/deg/information.php?ac=DEG10010191&db=p) |
| 10 | Cp1002_0228 | DNA polymerase III subunit gamma/tau | [DEG10170021;](http://tubic.tju.edu.cn/deg/information.php?ac=DEG10170021&db=p)[DEG10160163;](http://tubic.tju.edu.cn/deg/information.php?ac=DEG10160163&db=p)[DEG10170024;](http://tubic.tju.edu.cn/deg/information.php?ac=DEG10170024&db=p)[DEG10030223;](http://tubic.tju.edu.cn/deg/information.php?ac=DEG10030223&db=p)[DEG10220190;](http://tubic.tju.edu.cn/deg/information.php?ac=DEG10220190&db=p)[DEG10160114;](http://tubic.tju.edu.cn/deg/information.php?ac=DEG10160114&db=p)[DEG10330116;](http://tubic.tju.edu.cn/deg/information.php?ac=DEG10330116&db=p)[DEG10130274;](http://tubic.tju.edu.cn/deg/information.php?ac=DEG10130274&db=p)[DEG10060340;](http://tubic.tju.edu.cn/deg/information.php?ac=DEG10060340&db=p)[DEG10280012;](http://tubic.tju.edu.cn/deg/information.php?ac=DEG10280012&db=p)[DEG10250711;](http://tubic.tju.edu.cn/deg/information.php?ac=DEG10250711&db=p)[DEG10240273;](http://tubic.tju.edu.cn/deg/information.php?ac=DEG10240273&db=p)[DEG10320058;](http://tubic.tju.edu.cn/deg/information.php?ac=DEG10320058&db=p)[DEG10240256;](http://tubic.tju.edu.cn/deg/information.php?ac=DEG10240256&db=p)[DEG10140019;](http://tubic.tju.edu.cn/deg/information.php?ac=DEG10140019&db=p)[DEG10230084;](http://tubic.tju.edu.cn/deg/information.php?ac=DEG10230084&db=p)[DEG10210104;](http://tubic.tju.edu.cn/deg/information.php?ac=DEG10210104&db=p)[DEG10190054;](http://tubic.tju.edu.cn/deg/information.php?ac=DEG10190054&db=p)[DEG10020026;](http://tubic.tju.edu.cn/deg/information.php?ac=DEG10020026&db=p)[DEG10320116;](http://tubic.tju.edu.cn/deg/information.php?ac=DEG10320116&db=p)[DEG10070050;](http://tubic.tju.edu.cn/deg/information.php?ac=DEG10070050&db=p)[DEG10190092;](http://tubic.tju.edu.cn/deg/information.php?ac=DEG10190092&db=p)[DEG10330166;](http://tubic.tju.edu.cn/deg/information.php?ac=DEG10330166&db=p)[DEG10130241;](http://tubic.tju.edu.cn/deg/information.php?ac=DEG10130241&db=p)[DEG10290186;](http://tubic.tju.edu.cn/deg/information.php?ac=DEG10290186&db=p)[DEG10250726;](http://tubic.tju.edu.cn/deg/information.php?ac=DEG10250726&db=p)[DEG10180182;](http://tubic.tju.edu.cn/deg/information.php?ac=DEG10180182&db=p)[DEG10180088;](http://tubic.tju.edu.cn/deg/information.php?ac=DEG10180088&db=p)[DEG10010007;](http://tubic.tju.edu.cn/deg/information.php?ac=DEG10010007&db=p)[DEG10050150;](http://tubic.tju.edu.cn/deg/information.php?ac=DEG10050150&db=p)[DEG10120034;](http://tubic.tju.edu.cn/deg/information.php?ac=DEG10120034&db=p)[DEG10030388;](http://tubic.tju.edu.cn/deg/information.php?ac=DEG10030388&db=p)[DEG10200228;](http://tubic.tju.edu.cn/deg/information.php?ac=DEG10200228&db=p)[DEG10200037;](http://tubic.tju.edu.cn/deg/information.php?ac=DEG10200037&db=p)[DEG10270640;](http://tubic.tju.edu.cn/deg/information.php?ac=DEG10270640&db=p)[DEG10290244;](http://tubic.tju.edu.cn/deg/information.php?ac=DEG10290244&db=p)[DEG10070046;](http://tubic.tju.edu.cn/deg/information.php?ac=DEG10070046&db=p)[DEG10270651;](http://tubic.tju.edu.cn/deg/information.php?ac=DEG10270651&db=p)[DEG10280073;](http://tubic.tju.edu.cn/deg/information.php?ac=DEG10280073&db=p)[DEG10010009;](http://tubic.tju.edu.cn/deg/information.php?ac=DEG10010009&db=p)[DEG10210151;](http://tubic.tju.edu.cn/deg/information.php?ac=DEG10210151&db=p)[DEG10100587;](http://tubic.tju.edu.cn/deg/information.php?ac=DEG10100587&db=p) |
| 11 | Cp1002_1213 | Diphtheria toxin repressor | [DEG10250527;](http://tubic.tju.edu.cn/deg/information.php?ac=DEG10250527&db=p) |
| 12 | **Cp1002_0412^#^** | Alanine racemase | [DEG10070209;](http://tubic.tju.edu.cn/deg/information.php?ac=DEG10070209&db=p)[DEG10030067;](http://tubic.tju.edu.cn/deg/information.php?ac=DEG10030067&db=p)[DEG10250669;](http://tubic.tju.edu.cn/deg/information.php?ac=DEG10250669&db=p)[DEG10080161;](http://tubic.tju.edu.cn/deg/information.php?ac=DEG10080161&db=p)[DEG10270600;](http://tubic.tju.edu.cn/deg/information.php?ac=DEG10270600&db=p)[DEG10330338;](http://tubic.tju.edu.cn/deg/information.php?ac=DEG10330338&db=p)[DEG10230282;](http://tubic.tju.edu.cn/deg/information.php?ac=DEG10230282&db=p)[DEG10100540;](http://tubic.tju.edu.cn/deg/information.php?ac=DEG10100540&db=p)[DEG10220247;](http://tubic.tju.edu.cn/deg/information.php?ac=DEG10220247&db=p)[DEG10010072;](http://tubic.tju.edu.cn/deg/information.php?ac=DEG10010072&db=p)[DEG10050568;](http://tubic.tju.edu.cn/deg/information.php?ac=DEG10050568&db=p)[DEG10210054;](http://tubic.tju.edu.cn/deg/information.php?ac=DEG10210054&db=p)[DEG10290334;](http://tubic.tju.edu.cn/deg/information.php?ac=DEG10290334&db=p) |
| 13 | Cp1002_2084 | Anthranilate synthase component II | [DEG10270597;](http://tubic.tju.edu.cn/deg/information.php?ac=DEG10270597&db=p)[DEG10130295;](http://tubic.tju.edu.cn/deg/information.php?ac=DEG10130295&db=p)[DEG10270006;](http://tubic.tju.edu.cn/deg/information.php?ac=DEG10270006&db=p)[DEG10050500;](http://tubic.tju.edu.cn/deg/information.php?ac=DEG10050500&db=p)[DEG10250005;](http://tubic.tju.edu.cn/deg/information.php?ac=DEG10250005&db=p)[DEG10280368;](http://tubic.tju.edu.cn/deg/information.php?ac=DEG10280368&db=p)[DEG10250666;](http://tubic.tju.edu.cn/deg/information.php?ac=DEG10250666&db=p)[DEG10050421;](http://tubic.tju.edu.cn/deg/information.php?ac=DEG10050421&db=p)[DEG10100534;](http://tubic.tju.edu.cn/deg/information.php?ac=DEG10100534&db=p) |
| 14 | Cp1002_0436 | D-methionine-binding lipoprotein metQ | [DEG10020025;](http://tubic.tju.edu.cn/deg/information.php?ac=DEG10020025&db=p)[DEG10020076;](http://tubic.tju.edu.cn/deg/information.php?ac=DEG10020076&db=p) |
| 15 | Cp1002_0259 | Signal-transduction histidine kinase senX3 | [DEG10260016;](http://tubic.tju.edu.cn/deg/information.php?ac=DEG10260016&db=p)[DEG10270577;](http://tubic.tju.edu.cn/deg/information.php?ac=DEG10270577&db=p)[DEG10340402;](http://tubic.tju.edu.cn/deg/information.php?ac=DEG10340402&db=p)[DEG10300039;](http://tubic.tju.edu.cn/deg/information.php?ac=DEG10300039&db=p)[DEG10010262;](http://tubic.tju.edu.cn/deg/information.php?ac=DEG10010262&db=p)[DEG10270175;](http://tubic.tju.edu.cn/deg/information.php?ac=DEG10270175&db=p)[DEG10260096;](http://tubic.tju.edu.cn/deg/information.php?ac=DEG10260096&db=p)[DEG10200128;](http://tubic.tju.edu.cn/deg/information.php?ac=DEG10200128&db=p)[DEG10250166;](http://tubic.tju.edu.cn/deg/information.php?ac=DEG10250166&db=p)[DEG10300095;](http://tubic.tju.edu.cn/deg/information.php?ac=DEG10300095&db=p)[DEG10200087;](http://tubic.tju.edu.cn/deg/information.php?ac=DEG10200087&db=p)[DEG10130463;](http://tubic.tju.edu.cn/deg/information.php?ac=DEG10130463&db=p)[DEG10260069;](http://tubic.tju.edu.cn/deg/information.php?ac=DEG10260069&db=p)[DEG10200422;](http://tubic.tju.edu.cn/deg/information.php?ac=DEG10200422&db=p)[DEG10170010;](http://tubic.tju.edu.cn/deg/information.php?ac=DEG10170010&db=p)[DEG10250640;](http://tubic.tju.edu.cn/deg/information.php?ac=DEG10250640&db=p)[DEG10180268;](http://tubic.tju.edu.cn/deg/information.php?ac=DEG10180268&db=p)[DEG10100517;](http://tubic.tju.edu.cn/deg/information.php?ac=DEG10100517&db=p)[DEG10250729;](http://tubic.tju.edu.cn/deg/information.php?ac=DEG10250729&db=p)[DEG10200130;](http://tubic.tju.edu.cn/deg/information.php?ac=DEG10200130&db=p)[DEG10250179;](http://tubic.tju.edu.cn/deg/information.php?ac=DEG10250179&db=p)[DEG10110029;](http://tubic.tju.edu.cn/deg/information.php?ac=DEG10110029&db=p)[DEG10030697;](http://tubic.tju.edu.cn/deg/information.php?ac=DEG10030697&db=p)[DEG10100147;](http://tubic.tju.edu.cn/deg/information.php?ac=DEG10100147&db=p) |
| 16 | **Cp1002_0374^#^** | 50S ribosomal protein L30 | [DEG10290050;](http://tubic.tju.edu.cn/deg/information.php?ac=DEG10290050&db=p)[DEG10340463;](http://tubic.tju.edu.cn/deg/information.php?ac=DEG10340463&db=p)[DEG10220403;](http://tubic.tju.edu.cn/deg/information.php?ac=DEG10220403&db=p)[DEG10330322;](http://tubic.tju.edu.cn/deg/information.php?ac=DEG10330322&db=p)[DEG10170316;](http://tubic.tju.edu.cn/deg/information.php?ac=DEG10170316&db=p)[DEG10010053;](http://tubic.tju.edu.cn/deg/information.php?ac=DEG10010053&db=p)[DEG10050283;](http://tubic.tju.edu.cn/deg/information.php?ac=DEG10050283&db=p)[DEG10320271;](http://tubic.tju.edu.cn/deg/information.php?ac=DEG10320271&db=p)[DEG10030519;](http://tubic.tju.edu.cn/deg/information.php?ac=DEG10030519&db=p)[DEG10190211;](http://tubic.tju.edu.cn/deg/information.php?ac=DEG10190211&db=p)[DEG10020260;](http://tubic.tju.edu.cn/deg/information.php?ac=DEG10020260&db=p)[DEG10100120;](http://tubic.tju.edu.cn/deg/information.php?ac=DEG10100120&db=p)[DEG10110182;](http://tubic.tju.edu.cn/deg/information.php?ac=DEG10110182&db=p)[DEG10160318;](http://tubic.tju.edu.cn/deg/information.php?ac=DEG10160318&db=p) |
| 17 | **Cp1002_1592^#^** | Bifunctional protein folC | [DEG10240379;](http://tubic.tju.edu.cn/deg/information.php?ac=DEG10240379&db=p)[DEG10190135;](http://tubic.tju.edu.cn/deg/information.php?ac=DEG10190135&db=p)[DEG10330072;](http://tubic.tju.edu.cn/deg/information.php?ac=DEG10330072&db=p)[DEG10170239;](http://tubic.tju.edu.cn/deg/information.php?ac=DEG10170239&db=p)[DEG10340325;](http://tubic.tju.edu.cn/deg/information.php?ac=DEG10340325&db=p)[DEG10160070;](http://tubic.tju.edu.cn/deg/information.php?ac=DEG10160070&db=p)[DEG10270448;](http://tubic.tju.edu.cn/deg/information.php?ac=DEG10270448&db=p)[DEG10100395;](http://tubic.tju.edu.cn/deg/information.php?ac=DEG10100395&db=p)[DEG10210035;](http://tubic.tju.edu.cn/deg/information.php?ac=DEG10210035&db=p)[DEG10200434;](http://tubic.tju.edu.cn/deg/information.php?ac=DEG10200434&db=p)[DEG10130105;](http://tubic.tju.edu.cn/deg/information.php?ac=DEG10130105&db=p)[DEG10280459;](http://tubic.tju.edu.cn/deg/information.php?ac=DEG10280459&db=p)[DEG10030211;](http://tubic.tju.edu.cn/deg/information.php?ac=DEG10030211&db=p)[DEG10320189;](http://tubic.tju.edu.cn/deg/information.php?ac=DEG10320189&db=p)[DEG10230069;](http://tubic.tju.edu.cn/deg/information.php?ac=DEG10230069&db=p)[DEG10250478;](http://tubic.tju.edu.cn/deg/information.php?ac=DEG10250478&db=p)[DEG10220311;](http://tubic.tju.edu.cn/deg/information.php?ac=DEG10220311&db=p)[DEG10070126;](http://tubic.tju.edu.cn/deg/information.php?ac=DEG10070126&db=p)[DEG10290271;](http://tubic.tju.edu.cn/deg/information.php?ac=DEG10290271&db=p) |
| 18 | **Cp1002_1239^#^** | Diaminopimelate epimerase | [DEG10180553;](http://tubic.tju.edu.cn/deg/information.php?ac=DEG10180553&db=p)[DEG10270491;](http://tubic.tju.edu.cn/deg/information.php?ac=DEG10270491&db=p)[DEG10280320;](http://tubic.tju.edu.cn/deg/information.php?ac=DEG10280320&db=p)[DEG10050253;](http://tubic.tju.edu.cn/deg/information.php?ac=DEG10050253&db=p)[DEG10290376;](http://tubic.tju.edu.cn/deg/information.php?ac=DEG10290376&db=p)[DEG10330252;](http://tubic.tju.edu.cn/deg/information.php?ac=DEG10330252&db=p)[DEG10160249;](http://tubic.tju.edu.cn/deg/information.php?ac=DEG10160249&db=p)[DEG10340314;](http://tubic.tju.edu.cn/deg/information.php?ac=DEG10340314&db=p)[DEG10010231;](http://tubic.tju.edu.cn/deg/information.php?ac=DEG10010231&db=p)[DEG10100431;](http://tubic.tju.edu.cn/deg/information.php?ac=DEG10100431&db=p)[DEG10200460;](http://tubic.tju.edu.cn/deg/information.php?ac=DEG10200460&db=p)[DEG10250529;](http://tubic.tju.edu.cn/deg/information.php?ac=DEG10250529&db=p)[DEG10130331;](http://tubic.tju.edu.cn/deg/information.php?ac=DEG10130331&db=p) |
| 19 | **Cp1002_1270^#^** | Riboflavin biosynthesis protein ribF | [DEG10330002;](http://tubic.tju.edu.cn/deg/information.php?ac=DEG10330002&db=p)[DEG10130007;](http://tubic.tju.edu.cn/deg/information.php?ac=DEG10130007&db=p)[DEG10160002;](http://tubic.tju.edu.cn/deg/information.php?ac=DEG10160002&db=p)[DEG10170167;](http://tubic.tju.edu.cn/deg/information.php?ac=DEG10170167&db=p)[DEG10230136;](http://tubic.tju.edu.cn/deg/information.php?ac=DEG10230136&db=p)[DEG10080197;](http://tubic.tju.edu.cn/deg/information.php?ac=DEG10080197&db=p)[DEG10240063;](http://tubic.tju.edu.cn/deg/information.php?ac=DEG10240063&db=p)[DEG10220205;](http://tubic.tju.edu.cn/deg/information.php?ac=DEG10220205&db=p)[DEG10340505;](http://tubic.tju.edu.cn/deg/information.php?ac=DEG10340505&db=p)[DEG10210100;](http://tubic.tju.edu.cn/deg/information.php?ac=DEG10210100&db=p)[DEG10270501;](http://tubic.tju.edu.cn/deg/information.php?ac=DEG10270501&db=p)[DEG10290307;](http://tubic.tju.edu.cn/deg/information.php?ac=DEG10290307&db=p)[DEG10200098;](http://tubic.tju.edu.cn/deg/information.php?ac=DEG10200098&db=p)[DEG10180005;](http://tubic.tju.edu.cn/deg/information.php?ac=DEG10180005&db=p)[DEG10250541;](http://tubic.tju.edu.cn/deg/information.php?ac=DEG10250541&db=p)[DEG10280410;](http://tubic.tju.edu.cn/deg/information.php?ac=DEG10280410&db=p)[DEG10320004;](http://tubic.tju.edu.cn/deg/information.php?ac=DEG10320004&db=p)[DEG10100439;](http://tubic.tju.edu.cn/deg/information.php?ac=DEG10100439&db=p)[DEG10030146;](http://tubic.tju.edu.cn/deg/information.php?ac=DEG10030146&db=p)[DEG10120109;](http://tubic.tju.edu.cn/deg/information.php?ac=DEG10120109&db=p)[DEG10190001;](http://tubic.tju.edu.cn/deg/information.php?ac=DEG10190001&db=p) |
| 20 | Cp1002_1982 | LytR family transcriptional regulator | [DEG10170179;](http://tubic.tju.edu.cn/deg/information.php?ac=DEG10170179&db=p)[DEG10100523;](http://tubic.tju.edu.cn/deg/information.php?ac=DEG10100523&db=p)[DEG10250650;](http://tubic.tju.edu.cn/deg/information.php?ac=DEG10250650&db=p)[DEG10250682;](http://tubic.tju.edu.cn/deg/information.php?ac=DEG10250682&db=p) |
| 21 | Cp1002_0846 | ATP synthase gamma chain | [DEG10130027;](http://tubic.tju.edu.cn/deg/information.php?ac=DEG10130027&db=p)[DEG10240357;](http://tubic.tju.edu.cn/deg/information.php?ac=DEG10240357&db=p)[DEG10140096;](http://tubic.tju.edu.cn/deg/information.php?ac=DEG10140096&db=p)[DEG10080207;](http://tubic.tju.edu.cn/deg/information.php?ac=DEG10080207&db=p)[DEG10290396;](http://tubic.tju.edu.cn/deg/information.php?ac=DEG10290396&db=p)[DEG10200417;](http://tubic.tju.edu.cn/deg/information.php?ac=DEG10200417&db=p)[DEG10100206;](http://tubic.tju.edu.cn/deg/information.php?ac=DEG10100206&db=p)[DEG10060329;](http://tubic.tju.edu.cn/deg/information.php?ac=DEG10060329&db=p)[DEG10250244;](http://tubic.tju.edu.cn/deg/information.php?ac=DEG10250244&db=p)[DEG10270238;](http://tubic.tju.edu.cn/deg/information.php?ac=DEG10270238&db=p)[DEG10030560;](http://tubic.tju.edu.cn/deg/information.php?ac=DEG10030560&db=p)[DEG10280103;](http://tubic.tju.edu.cn/deg/information.php?ac=DEG10280103&db=p)[DEG10070183;](http://tubic.tju.edu.cn/deg/information.php?ac=DEG10070183&db=p)[DEG10210079;](http://tubic.tju.edu.cn/deg/information.php?ac=DEG10210079&db=p)[DEG10120358;](http://tubic.tju.edu.cn/deg/information.php?ac=DEG10120358&db=p) |
| 22 | Cp1002_0679 | Methionine--tRNA ligase | [DEG10160148;](http://tubic.tju.edu.cn/deg/information.php?ac=DEG10160148&db=p)[DEG10330151;](http://tubic.tju.edu.cn/deg/information.php?ac=DEG10330151&db=p)[DEG10320184;](http://tubic.tju.edu.cn/deg/information.php?ac=DEG10320184&db=p)[DEG10170025;](http://tubic.tju.edu.cn/deg/information.php?ac=DEG10170025&db=p)[DEG10010199;](http://tubic.tju.edu.cn/deg/information.php?ac=DEG10010199&db=p)[DEG10010010;](http://tubic.tju.edu.cn/deg/information.php?ac=DEG10010010&db=p)[DEG10020186;](http://tubic.tju.edu.cn/deg/information.php?ac=DEG10020186&db=p)[DEG10100151;](http://tubic.tju.edu.cn/deg/information.php?ac=DEG10100151&db=p)[DEG10140185;](http://tubic.tju.edu.cn/deg/information.php?ac=DEG10140185&db=p)[DEG10320072;](http://tubic.tju.edu.cn/deg/information.php?ac=DEG10320072&db=p)[DEG10220171;](http://tubic.tju.edu.cn/deg/information.php?ac=DEG10220171&db=p)[DEG10050332;](http://tubic.tju.edu.cn/deg/information.php?ac=DEG10050332&db=p)[DEG10180113;](http://tubic.tju.edu.cn/deg/information.php?ac=DEG10180113&db=p)[DEG10330081;](http://tubic.tju.edu.cn/deg/information.php?ac=DEG10330081&db=p)[DEG10130122;](http://tubic.tju.edu.cn/deg/information.php?ac=DEG10130122&db=p)[DEG10290105;](http://tubic.tju.edu.cn/deg/information.php?ac=DEG10290105&db=p)[DEG10190066;](http://tubic.tju.edu.cn/deg/information.php?ac=DEG10190066&db=p)[DEG10170240;](http://tubic.tju.edu.cn/deg/information.php?ac=DEG10170240&db=p)[DEG10010218;](http://tubic.tju.edu.cn/deg/information.php?ac=DEG10010218&db=p)[DEG10140025;](http://tubic.tju.edu.cn/deg/information.php?ac=DEG10140025&db=p)[DEG10250186;](http://tubic.tju.edu.cn/deg/information.php?ac=DEG10250186&db=p)[DEG10200182;](http://tubic.tju.edu.cn/deg/information.php?ac=DEG10200182&db=p)[DEG10050455;](http://tubic.tju.edu.cn/deg/information.php?ac=DEG10050455&db=p)[DEG10120117;](http://tubic.tju.edu.cn/deg/information.php?ac=DEG10120117&db=p)[DEG10270182;](http://tubic.tju.edu.cn/deg/information.php?ac=DEG10270182&db=p)[DEG10060013;](http://tubic.tju.edu.cn/deg/information.php?ac=DEG10060013&db=p)[DEG10230180;](http://tubic.tju.edu.cn/deg/information.php?ac=DEG10230180&db=p)[DEG10220048;](http://tubic.tju.edu.cn/deg/information.php?ac=DEG10220048&db=p)[DEG10160079;](http://tubic.tju.edu.cn/deg/information.php?ac=DEG10160079&db=p)[DEG10180342;](http://tubic.tju.edu.cn/deg/information.php?ac=DEG10180342&db=p)[DEG10190129;](http://tubic.tju.edu.cn/deg/information.php?ac=DEG10190129&db=p)[DEG10110126;](http://tubic.tju.edu.cn/deg/information.php?ac=DEG10110126&db=p)[DEG10020031;](http://tubic.tju.edu.cn/deg/information.php?ac=DEG10020031&db=p)[DEG10210150;](http://tubic.tju.edu.cn/deg/information.php?ac=DEG10210150&db=p)[DEG10290247;](http://tubic.tju.edu.cn/deg/information.php?ac=DEG10290247&db=p)[DEG10280074;](http://tubic.tju.edu.cn/deg/information.php?ac=DEG10280074&db=p) |
| 23 | Cp1002_0917 | Ribosomal RNA small subunit methyltransferase D | [DEG10280275;](http://tubic.tju.edu.cn/deg/information.php?ac=DEG10280275&db=p)[DEG10290385;](http://tubic.tju.edu.cn/deg/information.php?ac=DEG10290385&db=p) |
| 24 | Cp1002_1800 | Aerobic C4-dicarboxylate transport protein | [DEG10130260;](http://tubic.tju.edu.cn/deg/information.php?ac=DEG10130260&db=p)[DEG10300114;](http://tubic.tju.edu.cn/deg/information.php?ac=DEG10300114&db=p)[DEG10300028;](http://tubic.tju.edu.cn/deg/information.php?ac=DEG10300028&db=p) |
| 25 | Cp1002_0199 | Uncharacterized protein yqeY | [DEG10030099;](http://tubic.tju.edu.cn/deg/information.php?ac=DEG10030099&db=p) |
| 26 | **Cp1002_0001^#^** | Chromosomal replication initiator protein DnaA | [DEG10290267;](http://tubic.tju.edu.cn/deg/information.php?ac=DEG10290267&db=p)[DEG10340394;](http://tubic.tju.edu.cn/deg/information.php?ac=DEG10340394&db=p)[DEG10170001;](http://tubic.tju.edu.cn/deg/information.php?ac=DEG10170001&db=p)[DEG10270001;](http://tubic.tju.edu.cn/deg/information.php?ac=DEG10270001&db=p)[DEG10050351;](http://tubic.tju.edu.cn/deg/information.php?ac=DEG10050351&db=p)[DEG10060380;](http://tubic.tju.edu.cn/deg/information.php?ac=DEG10060380&db=p)[DEG10020001;](http://tubic.tju.edu.cn/deg/information.php?ac=DEG10020001&db=p)[DEG10120001;](http://tubic.tju.edu.cn/deg/information.php?ac=DEG10120001&db=p)[DEG10200005;](http://tubic.tju.edu.cn/deg/information.php?ac=DEG10200005&db=p)[DEG10180542;](http://tubic.tju.edu.cn/deg/information.php?ac=DEG10180542&db=p)[DEG10180379;](http://tubic.tju.edu.cn/deg/information.php?ac=DEG10180379&db=p)[DEG10280001;](http://tubic.tju.edu.cn/deg/information.php?ac=DEG10280001&db=p)[DEG10320309;](http://tubic.tju.edu.cn/deg/information.php?ac=DEG10320309&db=p)[DEG10250001;](http://tubic.tju.edu.cn/deg/information.php?ac=DEG10250001&db=p)[DEG10210001;](http://tubic.tju.edu.cn/deg/information.php?ac=DEG10210001&db=p)[DEG10140001;](http://tubic.tju.edu.cn/deg/information.php?ac=DEG10140001&db=p)[DEG10290004;](http://tubic.tju.edu.cn/deg/information.php?ac=DEG10290004&db=p)[DEG10030003;](http://tubic.tju.edu.cn/deg/information.php?ac=DEG10030003&db=p)[DEG10240351;](http://tubic.tju.edu.cn/deg/information.php?ac=DEG10240351&db=p)[DEG10150001;](http://tubic.tju.edu.cn/deg/information.php?ac=DEG10150001&db=p)[DEG10230112;](http://tubic.tju.edu.cn/deg/information.php?ac=DEG10230112&db=p)[DEG10150237;](http://tubic.tju.edu.cn/deg/information.php?ac=DEG10150237&db=p)[DEG10160275;](http://tubic.tju.edu.cn/deg/information.php?ac=DEG10160275&db=p)[DEG10010001;](http://tubic.tju.edu.cn/deg/information.php?ac=DEG10010001&db=p)[DEG10220001;](http://tubic.tju.edu.cn/deg/information.php?ac=DEG10220001&db=p)[DEG10330279;](http://tubic.tju.edu.cn/deg/information.php?ac=DEG10330279&db=p)[DEG10100001;](http://tubic.tju.edu.cn/deg/information.php?ac=DEG10100001&db=p)[DEG10110141;](http://tubic.tju.edu.cn/deg/information.php?ac=DEG10110141&db=p)[DEG10190255;](http://tubic.tju.edu.cn/deg/information.php?ac=DEG10190255&db=p) |
| 27 | Cp1002_2094 | Uncharacterized protein | [DEG10180441;](http://tubic.tju.edu.cn/deg/information.php?ac=DEG10180441&db=p) |
| 28 | Cp1002_0139 | Zinc-binding alcohol dehydrogenase | [DEG10180338;](http://tubic.tju.edu.cn/deg/information.php?ac=DEG10180338&db=p) |
| 29 | Cp1002_0775 | Aromatic amino acid transport protein | [DEG10170247;](http://tubic.tju.edu.cn/deg/information.php?ac=DEG10170247&db=p)[DEG10080173;](http://tubic.tju.edu.cn/deg/information.php?ac=DEG10080173&db=p) |
| 30 | Cp1002_1670 | Cytochrome C oxidase polypeptide I | [DEG10270553;](http://tubic.tju.edu.cn/deg/information.php?ac=DEG10270553&db=p)[DEG10250606;](http://tubic.tju.edu.cn/deg/information.php?ac=DEG10250606&db=p)[DEG10020088;](http://tubic.tju.edu.cn/deg/information.php?ac=DEG10020088&db=p)[DEG10100496;](http://tubic.tju.edu.cn/deg/information.php?ac=DEG10100496&db=p) |
| 31 | Cp1002_1276 | Ribosome-binding factor A | [DEG10020138;](http://tubic.tju.edu.cn/deg/information.php?ac=DEG10020138&db=p)[DEG10130060;](http://tubic.tju.edu.cn/deg/information.php?ac=DEG10130060&db=p)[DEG10160221;](http://tubic.tju.edu.cn/deg/information.php?ac=DEG10160221&db=p)[DEG10330224;](http://tubic.tju.edu.cn/deg/information.php?ac=DEG10330224&db=p)[DEG10120364;](http://tubic.tju.edu.cn/deg/information.php?ac=DEG10120364&db=p)[DEG10030136;](http://tubic.tju.edu.cn/deg/information.php?ac=DEG10030136&db=p)[DEG10250548;](http://tubic.tju.edu.cn/deg/information.php?ac=DEG10250548&db=p) |

^#^The bold highlighted reference protein loci are the essential and non-host homologous bacterial protein already reported as drug target in other pathogenic microorganisms.

**Table S5, additional file 5: List of other important 22 druggable but** not yet reported as putative targets were also searched for molecular functions, biological processes, cellular compartmentalisations and metabolic pathway roles.

| **S. No** | **Gene Symbol** | **Official full name** | **Function** | **Cellular component** | **Pathways** | **DEG Homology** | **Host Homology** | **Drug Target** |
| --- | --- | --- | --- | --- | --- | --- | --- | --- |
| 1 | Cp1002_1381 | Transport YidE/  YbjL family protein | MF: Cation transmembrane transporter activity BP: Potassium ion transport | Integral plasma membrane | Putative Transport  Protein pathway | Yes | No | Nil |
| 2 | Cp1002_1795 | Hypoxanthine-guanine phosphoribosyltransferase | MF: Hypoxanthine phosphoribosyltransferase activity BP: Purine ribonucleoside salvage | Cytoplasm | Purine metabolism, Metabolic pathways, Biosynthesis of secondary metabolites | Yes | No | Nil |
| 3 | Cp1002_1072 | Ferrochelatase | MF: Ferrochelatase activity, metal ion binding BP: Heme biosynthetic process | Cytoplasm | Porphyrin and chlorophyll metabolism, Metabolic pathways, Biosynthesis of secondary metabolites. | Yes | No | Nil |
| 4 | Cp1002_1186 | Threonine--tRNA ligase | MF: ATP binding, metal ion binding,  Threonine-tRNA ligase activity BP: Threonyl-tRNA aminoacylation | Cytoplasm | Aminoacyl-tRNA biosynthesis | Yes | No | Nil |
| 5 | Cp1002_0370 | 30S ribosomal  protein S8 | MF: rRNA binding, structural constituent of ribosome BP: Translation | Ribosome | Ribosome | Yes | No | Nil |
| 6 | Cp1002_0861 | Cysteine desulfurase | MF: Catalytic activity,  Pyridoxal phosphate binding | Cytoplasmic | Thiamine metabolism, Metabolic  pathways, Sulphur relay system | Yes | No | Nil |
| 7 | Cp1002_0228 | DNA polymerase III  subunit gamma/tau | MF: 3'-5' exonuclease activity, DNA-directed DNA polymerase activity, nucleoside-triphosphatase activity, nucleotide binding | Cytoplasmic | Purine metabolism,  Pyrimidine metabolism, Metabolic pathways, DNA polymerase, Mismatch repair, Homologous recombination. | Yes | No | Nil |
| 8 | Cp1002_1213 | Diphtheria  toxin repressor | MF: DNA binding, sequence-specific DNA binding transcription factor activity, transition metal ion binding | Cytoplasmic | Transcriptional regulator | Yes | No | Nil |
| 9 | Cp1002_2084 | Anthranilate synthase component II | BP: Metabolic process | Cytoplasmic | Phenylalanine, tyrosine and tryptophan biosynthesis, Metabolic pathways, Biosynthesis of secondary metabolites, Biosynthesis of amino acids | Yes | No | Nil |
| 10 | Cp1002_0436 | D-methionine-binding lipoprotein metQ | Nil | Cytoplasmic/ membrane | ABC transporters | Yes | No | Nil |
| 11 | Cp1002_0259 | Signal-transduction  histidine kinase senX3 | MF: ATP binding,  Phosphorelay sensor kinase activity | Membrane | Two-component system | Yes | No | Nil |
| 12 | Cp1002_1982 | LytR family  transcriptional regulator | Nil | Extracellular, membrane, Cytoplasmic | Transcriptional regulator | Yes | No | Nil |
| 13 | Cp1002_0846 | ATP synthase gamma chain | MF: ATP binding, proton-transporting ATP synthase activity, rotational mechanism, proton-transporting ATPase activity, rotational mechanism BP: plasma membrane ATP synthesis coupled proton transport | [Plasma membrane, proton-transporting ATP synthase complex, catalytic core F](http://www.ebi.ac.uk/QuickGO/GTerm?id=GO:0005886) | Oxidative phosphorylation,  Metabolic pathways | Yes | No | Nil |
| 14 | Cp1002_0679 | Methionine--tRNA ligase | MF: ATP binding, metal ion binding,  Methionine-tRNA ligase activity BP: Methionyl-tRNA aminoacylation | Cytoplasm | Seleno compound metabolism,  Aminoacyl-tRNA biosynthesis | Yes | No | Nil |
| 15 | Cp1002_0917 | Ribosomal RNA small subunit methyltransferase D | MF: Methyltransferase activity, nucleic acid binding BP: rRNA methylation | Cytoplasmic | Ribosomal RNA small  subunit methyltransferase | Yes | No | Nil |
| 16 | Cp1002_1800 | Aerobic C4-dicarboxylate transport protein | MF: Sodium dicarboxylate  symporter activity | [Integral component of membrane](http://www.ebi.ac.uk/QuickGO/GTerm?id=GO:0016021) | Two-component system | Yes | No | Nil |
| 17 | Cp1002_0199 | Uncharacterized protein yqeY | MF: Carbon-nitrogen ligase activity,  with glutamine as amido-N-donor | Cytoplasmic | Hypothetical Protein | Yes | No | Nil |
| 18 | Cp1002_2094 | Uncharacterized protein | Nil | Extracellular, membrane, Cytoplasmic | Putative transcriptional  regulator | Yes | No | Nil |
| 19 | Cp1002_0139 | Zinc-binding alcohol dehydrogenase | MF: Oxidoreductase activity,  Zinc ion binding | Cytoplasmic | Zinc-binding alcohol  dehydrogenase | Yes | No | Nil |
| 20 | Cp1002_0775 | Aromatic amino acid transport protein | MF: amino acid transmembrane  transporter activity | [Integral component of membrane](http://www.ebi.ac.uk/QuickGO/GTerm?id=GO:0016021) | Aromatic amino acid  transport protein | Yes | No | Nil |
| 21 | Cp1002_1670 | Cytochrome C oxidase polypeptide I | MF: Oxidoreductase,  Cytochrome-c oxidase activity, Heme binding  BF: Aerobic respiration, electron transport chain | [Integral component of membrane, plasma membrane](http://www.ebi.ac.uk/QuickGO/GTerm?id=GO:0016021) | Oxidative phosphorylation,  Metabolic pathways | Yes | No | Nil |
| 22 | Cp1002_1276 | Ribosome-binding factor A | BP: rRNA processing | Cytoplasm | Ribosome-binding factor A | Yes | No | Nil |
